# Lecciones aprendidas en salud pública frente a la COVID-19: una mirada hacia futuras epidemias y pandemias

**DOI:** 10.7705/biomedica.6772

**Published:** 2022-10-31

**Authors:** Claudia Milena Cuéllar-Segura

**Affiliations:** 1 Médica epidemióloga, magíster en Ciencias Médicas; directora de Epidemiología y Demografía, Ministerio de Salud y Protección Social, Colombia (abril a agosto de 2022) Ministerio de Salud y Protección Social Colombia

La pandemia desencadenada por el virus SARS-CoV-2 que produce la COVID-19 -declarada por la Organización Mundial de la Salud (OMS) el 11 de marzo de 2020- es, sin duda, una de las crisis sanitarias más graves que ha afectado al mundo actual, solo equiparable a la devastadora pandemia del siglo XX, la gripe española, que cobró entre 40 y 50 millones de vidas^1^. Según la OMS, a corte de 13 de septiembre de 2022, en el mundo se han presentado 606’459.140 casos confirmados de COVID-19 y 6’495.110 fallecidos^2^. En Colombia, según el Sistema de Vigilancia en Salud Pública (Sivigila), se han registrado 6’304.317 de casos confirmados de COVID-19 y 141.708 fallecidos^3^. Sin embargo, estas cifras de morbilidad y mortalidad no son comparables con las de la gripe española, teniendo en cuenta los avances en el conocimiento científico, las nuevas tecnologías y las medidas de control de que se dispone actualmente, que se han podido usar en otras pandemias más recientes (influenza AH1N1, MERS-CoV, sarampión, virus de chikunguña y virus de Zika).

Desde el inicio de la pandemia por COVID-19, se han adquirido diferentes aprendizajes en cuanto a su aparición, ecología, geografía y población afectada. No obstante, sí se presentan similitudes con eventos pasados en cuanto a las consecuencias en temas como la gobernanza y la gobernabilidad, la salud pública, la economía y el impacto social, entre otros aspectos. Los efectos y las secuelas solo se pueden constatar a mediano y largo plazo.

Es así que cuando la OMS/OPS emite las primeras alertas epidemiológicas sobre el coronavirus, los Estados Miembro -bajo el Reglamento Sanitario Internacional (RSI, 2005)- ya estaban llevando a cabo las evaluaciones del riesgo que les permitieran establecer la posible afectación de la población del país^4^. Con base en lo anterior y bajo este análisis, es que los países, incluido Colombia, revisan las capacidades básicas en materia de salud y gestión pública para establecer la primera fase de preparación en respuesta ante una situación que, según su magnitud, el director de la OMS la clasifica como un evento de salud pública de importancia internacional. Esto significa que, de esta manera, se exhorta e insta a todos los Estados Miembro a tener una respuesta internacional coordinada y proporcionada para evitar o mitigar su propagación.

Esta pandemia ya preveía un enorme impacto en la salud pública: era un virus nuevo que producía una enfermedad infecciosa, de fácil transmisión de persona a persona, con la particularidad de que individuos asintomáticos podían contagiar a otros. Asimismo, el 100 % de la población era vulnerable en menor o mayor grado de compromiso en las manifestaciones clínicas, según la edad, enfermedades de base, inmunogenicidad y capacidad infecciosa. Además, las nuevas variantes y sublinajes que el virus sigue presentando, son cada vez más transmisibles, aunque con un componente de menor gravedad. Es de resaltar que los grupos poblacionales como los adultos mayores y las personas con inmunosupresión a causa de enfermedades de base, siguen teniendo el mayor riesgo de enfermar, de ingresar a las unidades de cuidado intensivo y de morir.

Las medidas no farmacológicas (lavado frecuente e higiene de manos, uso de tapabocas, ventilación de espacios cerrados, distanciamiento físico) junto con las cuarentenas, fueron los principales pilares de contención, con los cuales se buscaba mitigar la transmisión del virus y disminuir la presión sobre los servicios de salud.

En la fase de mitigación se necesitaron otras medidas, por lo que el país buscó participar en las primeras pruebas de vacunas contra el SARS-CoV-2. Los laboratorios productores de vacunas (los reconocidos y otros nuevos), junto con las universidades y científicos del mundo, se aliaron para probar diferentes plataformas; en tiempo récord, generaron la información para determinar la producción de varias vacunas, para que los países pudieran acceder a ellas estableciendo negociaciones bilaterales y que, los que no tuvieran recursos, las pudieran adquirir por los mecanismos que la OMS había dispuesto para ello.

Por medio del Fondo de Mitigación de Emergencias (FOME) creado por el gobierno nacional para financiar la emergencia sanitaria, Colombia logró adquirir dichas vacunas e iniciar una de las estrategias más complejas del último tiempo: el Plan Nacional de Vacunación, desarrollado en dos fases. Los objetivos de la primera fase fueron reducir la mortalidad y la incidencia de casos graves por este virus y proteger a los trabajadores de la salud de la exposición; la segunda tuvo como objetivo reducir el contagio para generar inmunidad de rebaño, siempre manteniendo las medidas de bioseguridad y autocuidado.

Estos desafíos significaron para el país una respuesta que sobrepasaba la capacidad institucional inicial frente a la vigilancia epidemiológica y de laboratorio, la respuesta operativa de los servicios de salud, el suficiente personal en salud para abordarla, así como los recursos y los insumos necesarios para atender la emergencia. Se requería que el gobierno nacional estableciera una estrategia articulada con sus respectivos responsables y, además, la comprensión del comportamiento de la epidemia por parte de los integrantes del Sistema General de Seguridad Social en Salud, los demás sectores y la comunidad en general.

Para fortalecer la estrategia gubernamental, se tuvieron en cuenta las experiencias del país durante los últimos 14 años al abordar otras pandemias y epidemias. No obstante, haciendo un balance de estos dos años y seis meses desde el inicio de esta pandemia, se observa que hubo aprendizajes específicos que aumentaron las capacidades, las cuales deben potencializarse para futuras emergencias en salud pública, en los siguientes aspectos.

1. Red pública nacional de laboratorios. Se debe establecer una red con personal entrenado, y con insumos y reactivos necesarios para practicar las pruebas que se requieran según el evento; en este caso en particular, fueron las moleculares, específicamente la PCR como prueba de referencia. Además, hay que ampliar esta red hacia laboratorios colaboradores, privados o universitarios.
2. Aumento de la capacidad de atención de los servicios de salud. Para la COVID-19, se hizo principalmente en las unidades de cuidados intensivos debido a la gravedad de la enfermedad. Se pasó de tener 5.000 a más de 13.000 camas disponibles y se pudieron adquirir los equipos necesarios, tener la infraestructura adecuada y habilitarla para resolver la demanda generada. Esta capacidad de expandir los servicios es la manera de responder si se llega a presentar una situación similar a la que ya hemos atravesado.
3. Contar con uno de los sistemas de vigilancia en salud pública más avanzados y apropiados para las entidades territoriales. La vigilancia genómica, considerada por la OPS como referente regional, y el desarrollo de modelos matemáticos para predecir el comportamiento de la pandemia, permitieron actuar con anticipación, establecer las políticas públicas y hacer los abordajes específicos en algunas de las regiones del país y durante los picos más complejos que se presentaron.
4. Medidas de autocuidado y bioseguridad. Este fue uno de los aprendizajes que más ayudó durante la pandemia, pues había ansiedad e incertidumbre, y no se avanzaba en el conocimiento sobre el comportamiento el virus. Las medidas adoptadas permitieron contener y mitigar la infección en sus picos con alta transmisibilidad debido a las diferentes variantes circulantes. Los programas de protección social tuvieron un reconocimiento internacional, porque protegieron y apoyaron a la población más vulnerable en los aspectos financieros para ayudar a reducir la desigualdad y la pobreza.
5. Reacción y toma de decisiones. El adquirir conocimiento científico, apoyarse en las asociaciones científicas y compartir evidencia con los demás países, incluso por medios virtuales y con el apoyo de quienes estaban afrontando la pandemia mediante cooperación internacional, fueron y serán a futuro formas de articular una respuesta para tomar decisiones.
6. Vacunación. En conjunto con las demás medidas no farmacológicas, esta fue una intervención costo-efectiva que nos permitió disminuir el impacto negativo de la COVID-19. El país alcanzó las metas propuestas, con base en la experiencia adquirida con un programa ampliado de inmunización fortalecido y reconocido internacionalmente. Se aprendió sobre las condiciones de la cadena de frío y el transporte de las vacunas ultracongeladas, que nunca se habían puesto en práctica en el mundo. La aplicación de cuatro veces más vacunas de las que en Colombia se aplican regularmente en un año, era uno de los mayores retos para lograr los objetivos propuestos, mientras transcurría la epidemia, aumentaban los casos y fallecían las personas.

Por otra parte, están las siguientes capacidades existentes y que, insisto, han sido un claro ejemplo de respuesta.

1. La articulación entre entidades nacionales, subnacionales y locales, mediante el reconocimiento de las condiciones geográficas, poblacionales y culturales, y los recursos físicos, financieros y humanos, para ejecutar la estrategia de manera coordinada, aplicando la normatividad, las directrices y los protocolos.
2. Capacitar permanentemente al personal de vigilancia epidemiológica, el cual debe detectar cualquier situación sospechosa para generar una alerta nacional sobre las diferentes vías de ingreso (aérea, marítima o terrestre) o cualquier evento poblacional para generar acciones inmediatas como notificación, análisis, control y seguimiento.
3. La comunicación del riesgo genera un cambio sustancial en el abordaje de la respuesta. En nuestro país, permitió llegar a la comunidad, los sectores y las entidades del Estado, generando cambios en su comportamiento. Se logró controlar la infodemia producida por diferentes medios y redes sociales; además, transmitir las decisiones que cambiaban de un momento a otro, y hacerlo en un lenguaje claro y apropiado para cada público. La comunicación es una de las intervenciones que no solo es útil en situaciones de emergencia, es una medida primordial para la salud pública en general.

Aunque no tenemos certeza sobre la duración de esta pandemia, ojalá su fin sea cada vez más próximo. Por lo pronto, se debe tener presente que estamos enfrentando situaciones de salud pública cada vez complejas, pero, que lo podemos hacer de manera simultánea y coordinada. Aplicar lo aprendido permitirá mejorar la expectativa de vida de las personas, y afrontar cambios demográficos y de indicadores de salud, porque, como lo expresé anteriormente, las secuelas de una pandemia o de una epidemia pueden llegar a ser catastróficas y de larga duración. Esperemos que lo aprendido se incorpore en el acervo de la salud pública, como práctica regular en instituciones públicas y privadas, tanto en la atención como en la gestión y la investigación.

## Footnotes

1
https://www.paho.org/es/quienes-somos/historia-ops/muerte-purpura-gran-gripe-1918

2
https://covid19.who.int/?gclid=EAIaIQobChMIs8WDmKWT-gIV14RaBR0iXgvuEAAYASABEgIO3_D_BwE

3
http://www.ins.gov.co/Noticias/Paginas/Coronavirus.aspx

4
https://www.minsalud.gov.co/sites/rid/Lists/BibliotecaDigital/RIDE/INEC/INTOR/reglamento-sanitario-internacional.pdf

